# Acceptance of Online Medical Websites: An Empirical Study in China

**DOI:** 10.3390/ijerph16060943

**Published:** 2019-03-15

**Authors:** Yuan Tang, Yu-Tao Yang, Yun-Fei Shao

**Affiliations:** 1 The School of Management and Economics, University of Electronic Science and Technology of China, Chengdu 611731, China; shaoyf@uestc.edu.cn; 2 School of Management, Sichuan University of Science & Engineering, Zigong 643000, China; 3 School of Economics and Management, Harbin Institute of Technology, Weihai 264200, China; 17s030169@stu.hit.edu.cn

**Keywords:** technology acceptance model, online medical websites, doctor–patient relationship, public health

## Abstract

As a new type of public health service product, online medical websites (OMWs) are becoming quite popular. OMWs can address patients’ basic medical problems remotely and give health guidance online. Compared to traditional hospitals, OMWs are more convenient and inexpensive, they can usually provide a better service for patients with poor medical conditions (especially in rural areas), and they also contribute to the rational distribution of medical resources. Therefore, key factors that affect patients’ acceptance of OMWs must be identified to contribute to public health. By integrating perceived risk (PR) and the technology acceptance model (TAM), we proposed a modified TAM and clarified how PR and other factors affect patients’ behavioral intention (BI) towards OMWs. A sample of 245 research participants in China took part in this study and the structural equation model (SEM) was used to test our hypotheses. The results revealed that perceived usefulness (PU) is a positive predictor of BI but has no significant effect on attitude (ATT), while perceived ease of use (PEOU) can affect BI through PU and attitude (ATT). Moreover, trust (TRU) was identified as a mediator of PR and PU/PEOU. Also, the doctor–patient relationship (DPR) was shown to moderate PR and TRU. In order to increase patients’ BI, OMW providers need further innovations to improve patients’ TRU and reduce their PR.

## 1. Introduction

Public health issues have attracted attention from all around the world. Additionally, the ways to solve the problems of allocation of medical resources have become a matter of significance. Evidently, the problem of uneven medical allocation in developing countries is very prominent. In China, a developing country, the large population and scarce medical resources represent a contradiction that is difficult to eliminate, especially in Chinese rural areas [1]. As a new type of medical service to solve patients’ (“patients” in this text means “people that use or have experience of using OMWs”) basic medical problems remotely and provide a health consultation service online, Internet medical care gives us hope to eliminate the above contradiction. In recent years, with the support of the Chinese government [2,3,4], the Internet medical and health industry, represented by online medical websites (OMWs), has been developing rapidly. Currently, Chinese OMWs like Chunyuyisheng (www.chunyuyisheng.com) and Haodaifu Online (www.haodf.com) are providing services for countless patients with successful results. However, our pre-test with a sample of 100 research participants in 2018 indicated that only 21% of them have ever used OMWs, which means Chinese OMWs are yet to received wide acceptance among patients. Therefore, it is crucial to explore the key factors affecting patients’ behavioral intention (BI) towards OMWs in order to promote the rational distribution of medical resources and to help achieve greater public health.

The rise of Internet medicine in China is an inevitable result. Combining the reports of iResearch (iResearch Inc, Shanghai, China) and the actual situation in China, we summarize several reasons for this: (1) a surge in health-care needs caused by population aging [5] and the increasing in the prevalence and range of chronic diseases in China [6]; (2) inadequate supply of medical resources. The annual growth rate of the number of diagnosed and treated patients (8.52%) during 2004–2014 was much higher than that of certified/assistant physicians (3.69%) [7], and medical conditions in rural areas lag far behind cities [8,9]; (3) unbalanced allocation of medical resources. Hospitals in big cities attract a large number of patients by virtue of their popularity and well-known doctors, forming a siphonic effect [10]. The utilization rate of medical resources in developed cities is low, but the medical resources in rural areas are insufficient, so the problem of medical resource allocation appears [11]. OMWs, however, eliminate the limitations of time and space, allowing patients to access basic medical and health services through the Internet whenever and wherever. Consequently, promoting the OMWs will foster the Chinese medical and health industry.

Chinese OMWs have existed for over 10 years, but they have been used more frequently in recent years. For example, Haodaifu Online and Chunyuyisheng, which are two representative websites of OMWs, were built in 2006 and 2011. Nowadays, their daily average usage is around 200,000. OMWs allow patients to visit the websites at any time and in any place to seek basic medical help or health information from certified doctors, which is convenient and meets the needs of patients. This is illustrated by the example of Haodaifu Online, which contains more than 550,000 doctors in 9177 regular hospitals in China. If there are no top doctors in a patients’ area, or if a patient needs a professional diagnosis but he/she is too busy to go to a traditional hospital, he/she can register and then log into the website (www.haodf.com), choosing a type of a disease or finding the doctors he/she prefers. A doctor will diagnose him/her online after the patient pays a fee. Then, the doctor will give the patient a detailed report and provide a prescription for him/her to purchase a prescription drug at a nearby pharmacy. After receiving treatment, patients can also rate the doctor’s services to provide a reference for other patients. If a patient has diabetes, he can find contraindications directly in the diabetes column of the website and can even develop his own diet plan. In this respect, OMWs are more convenient and efficient than traditional hospitals according to patients who have tried them, because they can see a doctor at home, and they do not waste their time in the hospital registration line [12]. In addition, patients can get a doctor’s prescription from the website and then buy medicine from a pharmacy or online; in this way, the cost is much cheaper than traditional hospitals. Furthermore, patients in rural areas can communicate directly with well-known doctors without leaving home, which greatly alleviates the imbalance of medical resource allocation and contributes to greater Chinese public health. The aforementioned advantages can promote patients’ acceptance of OMWs; however, disadvantages still exist. First, with the method of online diagnosis and treatment, it is difficult to establish a good relationship between patients and doctors, which may affect patients’ trust in the website. Second, OMWs can only solve basic conditions; it is difficult to directly treat complex diseases. Finally, patients may perceive the use of OMWs to be risky, including financial risk, security risk, and time risk. These disadvantages will negatively affect patients’ acceptance of OMWs. Thus, we present a framework for integrating perceived risk (PR) and the technology acceptance model (TAM) to identify the key factors affecting patients’ behavioral intention (BI).

In this research, we want to test how perceived risk, trust, and the relationship between doctors and patients affects patients’ behavioral intentions towards OMWs if they are key influential factors. The cohort of this study is a reasonable size for the application of measures and to gain insights into causality, so it is suitable to apply the TAM framework and expand it to build our research model after reviewing relevant literature. To make our hypothesis more convincing, we conducted a survey and adopted an empirical approach to test our research model and to verify the hypotheses.

The rest of this paper is organized as follows. Section 2 presents a literature review which offers a short overview of OMWs, TAM, and PR. We present our research hypotheses and model in Section 3, followed by the explanation of the research methods in Section 4. The results obtained from our study are clarified in Section 5. Section 6 and Section 7 provide the discussion and conclusions of our study.

## 2. Literature Review

In this section, we present a literature review to allow readers to understand our problems comprehensively. In the first part, we clarify the origin, definitions, and characteristics of online medical websites, and we review the development process of the technology acceptance model (TAM) and some representative empirical studies that applied the TAM framework as the basis of their research model in part 2. In part 3, we review the definition and studies related to the doctor–patient relationship. We searched the literature (key words: “online medical website*”, “online medical*”, “smart health*”, “technology acceptance model*”, “TAM*”, “consumers’ adoption*”, “doctor–patient relation*”, “physician-patient relation*”) in Science Citation Index Expanded, Social Science Citation Index, and CNKI (the most authoritative database of academic papers in China), and articles were included if they were meaningful, transformative (for example, a literature that has made leaps and bounds in a particular field), and suitable for our theme.

### 2.1. Online Medical Websites (OMWs)

#### 2.1.1. Online Medical Services and Online Medical Websites

The Internet has become a worldwide infrastructure in people’s lives [13]. People can access a countless amount of information which can solve their problems easily and quickly from anywhere through a mobile terminal connected to the Internet [14]. For example, medical institutions and doctors can provide medical services on the Internet for patients to choose from, and patients can use these services to check information about their symptoms, ask doctors to diagnose the condition remotely and issue a prescription, and make an appointment at home ahead of time for registration or surgery schedules. These services are named online medical services.

Many international medical institutions have been experimenting with online medical services for a long time. The University Hospital of Zurich has provided an online medical consultation service since 1999 [15]. Online medical databases can provide medical and health knowledge for the elderly [16]. Recently, online medical services have also been extended to the Internet of Things (IoT) and big data [17,18,19]. In China, online medical services have matured, covering many aspects of the medical field. The PDiag scheme, presented by Liu et al. in 2016, which is an efficient and privacy-preserving medical primary diagnosis scheme [20] and relevant issues about online health communities have been brought to the fore [21]. Researchers have also considered patients’ feedback on online medical services by studying patients’ comments on online doctors to improve online medical services [22].

Jiang (2017) classified online medical services into five categories: medical portal websites, continuing medical education websites, medical e-commerce websites, medical communities/interest groups/forums, and online medical websites [23]. Among them, online medical websites are currently one of the most popular types in China. Their users are both doctors and patients; doctors use OMWs to offer medical advice to patients across the country and to earn fair pay, while patients can obtain services such as online registration, online payment, and medical and health consultations to solve their problems conveniently and quickly and save time and money. In addition, unlike the lack of customer feedback in traditional hospitals, patients on OMWs can freely hire doctors and evaluate them after receiving services, which can be a reference for other patients. This kind of mechanism can increase equality between doctors and patients, and the demand for online medical platforms can be increased by word-of-mouth [24]. Finally, OMWs can reconfigure medical resources reasonably and provide social support for online patients [25].

#### 2.1.2. The Characteristics of OMWs

OMWs are one of the most important online medical services, and they have become gradually more authoritative in recent years. Medical undergraduate degree holders can study for medical master programs including Master of Nursing, Master of Clinical Medicine and Master of Basic Medicine from Zhengzhou University through xuetangX (one of Chinese most representative MOOC (Massive Open Online Courses) platforms which was constructed by Tsinghua University, http://www.xuetangx.com); OMWs, such as Chunyuyisheng and Haodaifu, have officially cooperated with hundreds of third-grade class-A hospitals (top hospitals in China) in China to provide medical services for patients. Therefore, there is no doubt that the most prominent characteristic of OMWs is “online”—it means patients can remotely acquire the medical services they need anytime and anywhere.

However, Internet-based online services always bring various risks (like security risk) to their users [26]. Rosa (2003) defined risk as a situation or event in which something of human value (including people themselves) is at stake and in which the outcome is uncertain [27]. The actual risks do not necessarily affect users’ acceptance, because users may not have received the risk information or have received it but have no sense of resistance [28]. Therefore, we use perceived risk (PR) to describe the risk level that has been received by users that is expected to affect users’ acceptance. Perceived risk is generally considered to include mainly financial risk, performance risk, psychological risk, physical risk, social risk, and time risk [29,30]. According to the characteristics of OMWs and the results of a previous pre-test, we considered the main dimensions of PR that may affect patients’ acceptance of OMWs to include perceived privacy risk (PPriR), perceived financial risk (PFR), and perceived physical risk (PPhyR).

OMWs are often considered convenient, efficient [11], and patient-centered platforms, like a primary care provider [31]. Therefore, we believe that OMWs are like service providers that focus on meeting the need of patients. During this process, the relationship between doctors and patients may usually be a factor that is important but easily ignored. Furthermore, Christensen et al. (2010) believe that trust in websites largely determines which website is accessed and how information is utilized once a patient has chosen a website [32]. Therefore, gaining consumers’ trust is crucial for OMWs to gain success. In conclusion, we regard the main characteristics of OMWs as convenience, efficiency, online and risky, patient-centered, and trust-conducted. Finally, three factors (perceived risk, trust, and the doctor–patient relationship) were used as the three basic variables to construct our research model from these characteristics.

### 2.2. Modified Technology Acceptance Model (TAM)

The technology acceptance model (TAM) which originated from the theory of planned behavior (TPB) and theory of reasoned action (TRA) [33,34] is thought of as the primary authoritative model for researching users’ acceptance of a specific technological product [35,36]. Davis (1989) figured out that observed variables, which are also named external variables, can affect users’ attitudes (ATT) using through two mediators, perceived usefulness (PU) and perceived ease of use (PEOU), while ATT can affect actual system use indirectly through behavioral intention (BI) to use. Additionally, PEOU can affect PU, and PU can directly affect BI [37].

With the help of TAM, researchers can choose external variables that fit their research content to study users’ acceptance of a specified product. Hence, researchers have proposed many modified TAMs based on Davis’ TAM. Venkatesh and Davis (2000) presented TAM2 to enhance the explanation of TAM; they integrated social influence processes (subjective norm and image) and cognitive instrumental processes (job relevance, output quality, result demonstrability) as external variables and added experience and voluntariness as moderators to expand TAM [38]. After that, on the basis of summarizing the research on TAM over the years, Venkatesh et al. (2003) integrated TAM, task-technology fit, innovation diffusion theory, a motivational model, a model of PC utilization, and social cognitive theory to form the unified theory of acceptance and use of technology. They added performance expectancy, effort expectancy, social influences, and facilitating conditions as external variables, and gender, age, experience, and voluntariness of use were used as moderators [39]. However, the theory framework of the previous model is too complicated to explain users’ acceptance of different products in different cultural environments. Therefore, when studying patients’ acceptance of medical services, many researchers adopted TAM as a basic model and then added appropriate external variables, mediators, and moderators to form modified TAMs [40,41,42,43,44,45].

### 2.3. Doctor–Patient Relationship (DPR)

The doctor–patient relationship (DPR) is defined as “the extent of familiarity, trust, and interaction between doctors and patients in the context of healthcare planning” [35]. In China, issues related to the DPR have become a common topic that has been widely discussed due to the generally bad relationships between doctors and patients [46]. The main reasons affecting DPR in China include unbalanced allocation of medical resources, rising medical expenses, low level of medical services, and poor communication between doctors and patients [47]. Therefore, it is important to include DPR in the research framework when studying Chinese medical services.

A good DPR should be built if the medical quality is to be improved continuously [48,49]. Many studies have shown that the DPR affects users’ (doctors’ or patients’) intentions to adopt specified medical technology in some ways; this especially occurred after the Internet became a ubiquitous phenomenon in the DPR [50]. Abdekhoda (2015) studied physicians’ acceptance of electronic medical records and stated that DPR will directly and significantly affect physicians’ attitude to use OMWs [51]. Dou (2017) proposed that DPR will affect patients’ acceptance of smartphone health technology through affecting PEOU [52]. Nowadays, DPR is considered an important factor that affects patients’ acceptance of e-health systems [53]. However, the current body of literature has hardly considered that the DPR may play the role of moderator rather than an observed variable that directly affects intention to use OMWs during the process of patients’ accepting medical services. Considering that the strength of DPR may change patients’ psychological conditions so that the influences of observed variables on latent variables might change, we tried to consider DPR as a moderator in this paper.

## 3. Research Hypotheses and Model

**Figure 1:**
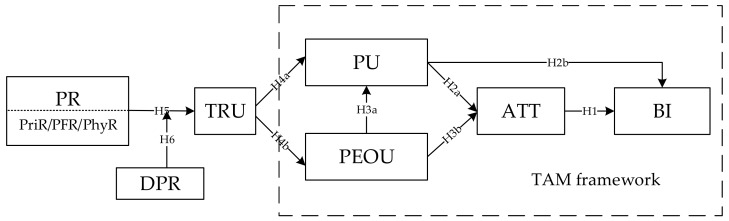
Research Model. (“H” in the model diagram refers to the research “hypothesis”).

Note: Perceived Usefulness (PU); Perceived Ease-of-Use (PEOU); Doctor–Patient Relationship (DPR); Perceived Risk (PR); Perceived Privacy Risk (PriR); Perceived Financial Risk (PFR); Perceived Physical Risk (PhyR); Attitude (ATT); Behavioral Intention (BI); Trust (TRU).

Based on the technology acceptance model (TAM), a theorical model, which is shown in Figure 1, was proposed to identify the key factors influencing patients’ acceptance of OMWs by combining the concept, characteristics, and doctor–patient relationship of OMWs. In our research model, PU, PEOU, ATT and BI are main factors of TAM, and our observed variable (external variable), named perceived risk (PR), contains three parts: PPriR, PFR, and PhyR. The basic assumption is that PR will affect PU and PEOU through the mediator TRU, and further, it will affect patients’ ATT and BI regarding the acceptance of OMWs. Additionally, DPR can play the role of moderator for the influence of PR on TRU.

### 3.1. TAM Framework

#### 3.1.1. Attitude (ATT) and Behavioral Intention (BI)

Attitude (ATT) is defined as “a learned predisposition to respond in a consistently favorable or unfavorable manner with respect to a given object” [54] while behavioral intention (BI) is defined as the subjective probability that an individual will perform a specified behavior [33,55], and many researchers have used intentions as a proxy for actual behaviors [56]. Therefore, we consider that if consumers show a positive/negative BI, they have a tendency to use/not use a specified product.

These two variables and their relationship were proposed by theory of planned behavior (TPB) [57], and ATT has been thought of the most powerful predictor of BI in the field of technology acceptance [58]. Numerous current studies consider ATT as having an indispensable role in determining BI when using TAM [59,60,61,62] according to Davis’ suggestion [33,34,55]. However, there are still doubts about this result, which has been proven many times. For example, Yang and Yoo (2005) proposed that when attitude is divided into cognitive attitude and affective attitude, only the former shows a direct influence on BI while the latter cannot affect BI significantly [63]. Therefore, it is meaningful for this study to test whether patients’ ATT can significantly affect their BI regarding OMWs. Thus, we proposed the following hypothesis:

**Hypothesis** **1.** ATT is positively related to patients’ BI.

#### 3.1.2. Perceived Usefulness (PU)

Perceived usefulness (PU) describes the degree to which people believe that using a particular system will enhance their job performance [37]. By combining the characteristics of OMWs, PU can describe the extent to which a patient believes that OMWs are useful for solving their problems.

Davis’ TAM suggests that PU will have direct influences on ATT and BI [33]. Although this viewpoint has been certified by numerous studies in recent years [61,64,65], many different opinions have been proposed. Some researchers believe that PU can affect BI through ATT but cannot affect BI directly [66,67]. Others think that PU can directly affect BI but have not considered the mediator role of ATT in this process [68,69,70]. We consider that the main reason for these differences is that the ability of PU to affect BI differs among users under different backgrounds. Therefore, in order to study the user acceptance of OMWs, it is necessary to determine the effects of PU on ATT and BI. Thus, we proposed the following hypotheses:

**Hypothesis** **2a.** PU is positively related to patients’ ATT.

**Hypothesis** **2b.** PU is positively related to patients’ BI.

#### 3.1.3. Perceived Ease of Use (PEOU)

Perceived ease of use (PEOU) refers to “the degree to which the prospective user expects the target system to be free of effort” in Davis’ TAM [33]. In the context of OMWs, PEOU can be defined as the extent to which a patient thinks that adopting OMWs will be free of effort. An example of PEOU is that patients believe that it is easy for them to understand how to use OMWs to solve their problems.

Current literature provides sufficient evidence to illustrate that PEOU has a positive effect on PU and ATT [65,68,71]. However, Liu (2005) did not find a significant effect of PEOU on either PU or BI [72]. In the field of medical services, PEOU has been certified as an important predictor of user acceptance. For example, Vassilios (2009) believed that PEOU influences the PU, ATT, and BI towards the use of information technology (IT) in hospitals [64]. Analogously, PEOU may have a positive effect on patients’ BI of using OMWs. Thus, we proposed the following hypotheses:

**Hypothesis** **3a.** PEOU is positively related to patients’ PU.

**Hypothesis** **3b.** PEOU is positively related to patients’ ATT.

### 3.2. Characteristics of OMWs

#### 3.2.1. Trust (TRU)

Trust (TRU), the catalyst between the buyer and the seller [73], is defined as “a psychological state comprising the intention to accept vulnerability based upon positive expectations of the intentions or behavior of another” [74].

Many researchers believe that TRU positively affects PU and PEOU. For example, when studying consumers’ adoption of electronic commerce, Pavlou (2003) figured out that TRU has significant positive effects on PU and PEOU, but he thought that perceived risk could be affected negatively by PU but not by the latter [73]. However, different researchers have different opinions. Beldad (2017) believes that TRU will be affected by PEOU although it can affect PU positively [75]. Anyway, TRU is usually used as an important factor which can affect consumers’ intention to use OMWs directly or indirectly [73,75,76]. Therefore, we believe that if patients have enough TRU in OMWs, they will find OMWs more useful and easier to use. Additionally, if the patients perceive that using OMWs will bring them some kinds of risk (financial risk, safety risk or privacy risk), they may not trust OMWs anymore and will not use them. Thus, we proposed the following hypotheses:

**Hypothesis** **4a.** TRU is positively related to patients’ PU.

**Hypothesis** **4b.** TRU is positively related to patients’ PEOU.

#### 3.2.2. Perceived Risk (PR)

Perceived risk (PR) refers to “an individual’s subjective evaluation of his or her risk of an illness or an adverse outcome, often in relation to performing a certain risky behavior” [77]. In this research, we consider that PR contains three main dimensions, which are named PPriR, PFR and PPhyR, based on OMWs’ characteristics. In the context of our research, PPriR, PFR, and PPhyR represent the differences between the loss due to possible privacy, financial, and physical risk, respectively, from using OMWs and the benefits they get from it. For example, patients may perceive PPhyR if they are concerned that the treatments from doctors of OMWs will negatively affect their health. Therefore, we believe that PR will negatively affect patients’ TRU of OMWs.

Although some researchers do not believe that PR could affect consumers’ intentions [78], there are still some research results that support our opinion. Wu and Ke (2015) used a meta-analysis to analyze a database containing three studies (1456 participants), and the results showed that the simple mean correlation between PR and TRU was significant [79]. Thus, we proposed the following hypothesis:

**Hypothesis** **5.** PR is negatively related to patients’ TRU.

#### 3.2.3. Doctor–Patient Relationship

The relationship between a doctor and a patient is usually based on a brief encounter during the process of treatment. As an important factor affecting this kind of relationship, the quality of communication may influence the doctor–patient relationship (DPR) [80]. In the field of medical services, DPR is defined as “the extent of familiarity, trust, and interaction between physicians and patients in the context of healthcare planning” [35]; therefore, we believe that the DPR could be a key factor in the research of how consumers build trust in medical services.

Some researchers have studied the ways in which the DPR affects BI. When studying patients’ adoption of a web-based personal health record system, Liu and Tsai (2013) certified that the DPR has a positive effect on patients’ behavioral intention to use the platform [35]. Dou et al. (2017) indicated that the DPR would affect BI through PU and PEOU [52]. Thus, the DPR is considered an important factor that can influence patients’ adoption of medical services. However, our opinion is different from current literature, as we do not think the DPR affects patients’ BI directly or indirectly. The actual situation that we observed is that many patients would not give up their chance of treatment for fear of not having a good relationship with their doctors. Therefore, in most cases, treatment or not would not be a problem for troubled patients; the real problem is which medical services provider should be chosen. Therefore, we suggest that DPR may play the role of moderator during consumers’ adoption of OMWs. When there is a harmonious relationship between the patient and the doctor, the DPR is good, and the negative effect of PR on TRU will be weakened; on the other hand, when the DPR is bad, the negative effect of PR on TRU will be strengthened. Thus, we proposed the following hypothesis:

**Hypothesis** **6.** DPR significantly moderates the relationship between PR and TRU.

## 4. Method

In this study, we adopted a survey approach to examine the hypotheses presented in the previous section because we needed to get original data from the target users of OMWs to certify whether our hypotheses could be supported by statistics or not. As online medical services are a kind of service formed by combining medical services and emerging Internet technology (IT), which was the basis for the birth and development of this industry, the research on patients’ acceptance of online medical services can be assessed with the technology acceptance model (TAM) [33]. In addition, as TAM alone cannot include all the features of a new product, researchers usually use TAM as a research framework [71,81,82,83]. Therefore, this study combined the TAM and the characteristics of OMWs to establish the research model and develop the questionnaire. The structural equation model (SEM) allows several endogenous variables to be considered at the same time as well as the existence of the measuring errors and residuals between exogenous variables and endogenous variables [84]. Moreover, the SEM allows researchers to clearly map out the relationship between multiple latent variables [85]. After comparison and analysis, we concluded that using the SEM to test our data was the most suitable method for this research [86]. Therefore, the SEM was employed to test the research model formulated in the previous sections. In addition, in order to improve the questionnaire, this study carried out a pre-test of 30 interviewees before the formal survey [87].

### 4.1. Questionnaire Development

We adopted a questionnaire with two sections to test our theoretical model [88]. Part 1 had 31 questions that were used to examine the constructs, and Part 2 was a personal information card that was used to gather some basic information about the interviewees. We presented a statement on each questionnaire to inform that there were no right/wrong or good/bad answers in the questionnaire and that all collected data were confidential and for academic use only.

Based on the characteristics of OMWs, the research model consisted of 7 constructs. Among them, perceived risk is composed of perceived privacy risk, perceived financial risk, and perceived safety risk, including 9 factors in total, and each factor was assessed by 2 to 5 items. In order to improve the content validity, most of the items were derived from the current literature [89]. To ensure that the meaning of the items could be understood clearly by researchers and interviewees, after the first researcher had translated the original items (English/Chinese), another researcher independently translated them into English/Chinese and then compared it with the original, until our questionnaire had the same meaning as the original and could accurately convey the characteristics of the OMWs. Finally, we completed a pre-test with 30 interviewees and modified part of the questionnaire content according to their feedback to form the final questionnaire.

The final version of the questionnaire had 34 items. There were 31 items in Part 1, covering 7 factors: PU, PEOU, ATT, BI, TRU, PR and DPR. Part 2 consisted of 3 items, including several basic characteristics of research participants like gender, age, and education. The factors and their items are shown in Appendix A. Each item in Part 1 corresponding to the constructs was measured using a 7-point Likert scale, where “1 means strongly disagree” and “7 means strongly agree”. Items for PU and PEOU were adapted from those of Venkatesh and Davis (2000) [38]; items for ATT and BI were adapted from Venkatesh et al. (2003) [39]; and items for DPR, TRU, PPriR, PFR, and PPhyR were adapted from those of Chung-Feng Liu et al. (2013) [35], Lee (2005) [90], Hajli and Lin (2016) [91], and Featherman and Pavlou (2003) [92], respectively. It should be noted that since PR contains three dimensions (PPriR, PFR, and PPhyR), each with 2–4 items, we calculated the average value of multiple items of each dimension as the value of that dimension during the process of data analysis. For example, PR had three items (PPriR, PFR, and PPhyR) and among them, PFR had two items (PFR1 and PFR2), so we calculated the average value of PFR1 and PFR2 as the value of PFR.

### 4.2. Data Collection

Our questionnaires were completed by adults (≥18 years old) who had experience with using online medical websites to ensure that interviewees could understand the content of the questionnaires accurately. To ensure that the survey was widely distributed, we collected data online and offline at the same time. We sent the questionnaire to Wenjuanxing (www.wenjuanxing.com), which is one of most widely used online questionnaire collection platforms in China, and then we distributed it through Wechat, which is one of the most popular social networks in China. The online questionnaires were openly available to any users of these services, but only users who met our requirements could fill out the questionnaires. As a result, we collected 132 responses online in total. Meanwhile, we collected data offline. Constrained by the resources of researchers, several cities (including Chengdu, Zigong, Xi’an, Weihai, Tsingtao, and Leshan) that were accessible to researchers were selected at first. Then, we selected 2 hospitals from each city by a random sampling method. Lastly, we looked for research participants in the waiting rooms of each hospital to fill in the questionnaires, and these were handed out by researchers personally. Since surgical and radiological services are not available on OMWs, all the respondents were from clinical departments. Anyway, we collected 136 copies offline. We also discussed the ethical concerns surrounding the inclusion of research participants in the research and the universal principles of informed consent. The participants had to satisfy the following criteria: (1) aged above or equal to 18 years; (2) agreed to be included in this research and filled out the questionnaire voluntarily; (3) participants were anonymous in order to protect their privacy; (4) participants were aware that there were no right/wrong or good/bad answers in this questionnaire and that all collected data were confidential and for academic use only; and (5) participants had to have experience with using online medical websites. And the authors confirm that this research has been carried out in accordance with Chinese ethical research rules.

We collected data from April to December in 2018; overall, 268 copies were returned. After excluding 23 responses that had duplicate IPs (internet protocols) or too many missing values, we had 245 valid responses in total. We collected the IPs of the respondents and only used them to eliminate the repeated questionnaires from duplicate IPs to ensure that each questionnaire was filled out by a different respondent. After doing this, the IP data were deleted from our database. The demographics of respondents are summarized in Table 1.

## 5. Results

There are three parts in this section, including the measurement model, structural equation model, and moderation effects. Using the two-step procedure suggested by Anderson and Gerbing (1988) [93], the reliability and validity of our scale and data are tested in the first part, and the second part shows the results of a path analysis for our research model. In order to determine the mechanism by which DPR can moderate PR’s effect on TRU, we conduct a regression analysis in the third part.

### 5.1. Measurement Model

We conducted three steps to examine the reliability, validity, and goodness of fit in this part. The data analysis results are shown in Table 2.

**Step 1**: The reliability of our collected data was examined with the statistical tool IBM SPSS Statistics 25. At first, we conducted several reliability analyses of all variables and the whole questionnaire. The results indicated that the Cronbach’s alpha of our seven constructs and the whole questionnaire were all greater than the recommended guideline of 0.70 [94], ranging from 0.78 (behavioral intention) to 0.89 (perceived risk). Then, we tried to conduct a factor analysis to estimate the factor loading and standard error of every item. Before that, we conducted the Kaiser–Meyer–Olkin (KMO) and Bartlett’s sphericity tests; the KMO of our data was 0.906 (>0.800), which means that our data are suitable for factor analysis. The factor analysis results showed that except PEOU1 (0.647), PEOU4 (0.671), ATT2 (0.661), and BI2 (0.595), all factor loadings of items were above 0.7, and they were all significant at the 0.05 level [95]. Last, we calculated the composite reliability (CR) score of each construct. The results showed that the CR score of each construct was higher than 0.7 [96]. Therefore, our data showed good reliability. The equation of CR is as follows:

$$CR=\frac{{\left(\Sigma \lambda_{i}\right)}^{2}}{{\left(\Sigma \lambda_{i}\right)}^{2}+\Sigma \Theta_{\mathrm{ii}}}\left(\lambda_{i}\;\mathrm{is}\;\mathrm{factor}\;\mathrm{loading},\;\Theta_{\mathrm{ii}}\;is\;\mathrm{the}\;\mathrm{standard}\;\mathrm{error}\right).$$

**Step 2:** The validity of our data was examined. First, we calculated the average variance extracted (AVE) of every construct. As shown in Table 2, all AVE values in this study were better than the recommended value of 0.5 [97], suggesting a good convergent validity. Then, we tried to examine the discriminant validity of constructs using a correlation analysis. We used Fornel and Larcker’s (1981) suggestion [95] that the average variance between a construct and its measurement should be greater than that of other constructs in our research model. As shown in Table 3, the square root of AVE (reported in the diagonal of correlation matrix) of each construct was higher than the correlation coefficients between that construct and other constructs, which suggested a good discriminant validity. The equation of AVE is as follows:

$$AVE=\frac{{\Sigma \lambda_{i}}^{2}}{{\Sigma \lambda_{i}}^{2}+\Sigma \Theta_{\mathrm{ii}}}\left(\lambda_{i}\;\mathrm{is}\;\mathrm{factor}\;\mathrm{loading},\;\Theta_{\mathrm{ii}}\;is\;\mathrm{the}\;\mathrm{standard}\;\mathrm{error}\right).$$

**Step 3:** The goodness of fit of our research model was examined. Considering that we needed to calculate the relationships between multiple constructs [98], a structural equation was adopted to test our modified TAM. Several key indices were evaluated to indicate the matching degree between our research model and the data. Table 4 summarizes the recommended and actual values of the fit indices, and all fit indices had more favorable values than the recommended ones [96,97,99]. Therefore, our research model has fit the data well.

### 5.2. Structural Equation Model

**Figure 2:**
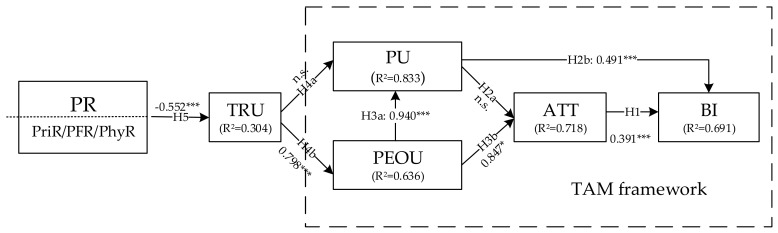
Path analysis (note. * *p* < 0.1; ** *p* < 0.05; *** *p* < 0.01).

To estimate the relationships between constructs properly and accurately, a structural equation model (SEM) was adopted to test our research hypotheses using IBM SPSS AMOS 22.0.0, and the results are shown in Figure 2 and Table 5.

Figure 2 shows that the comprehensive effect R^2^ value of BI was 0.691, which means 69.1% of the variance in BI was explained by the model. Thus, our research model can explain the majority of patients’ behavioral intention. The R^2^ values of TRU, PU, PEOU, and ATT reached 0.304, 0.833, 0.636, and 0.718, respectively, thus explaining 30.4%, 83.3%, 63.6%, and 71.8% of the variance in the corresponding constructs. As a result, all of the constructs in this research were well explained.

The path analysis results of our structural equation model are summarized in Table 5. Most of the paths were significantly in line with our expectation, except H2a (PU has a positive effect on ATT) and H4a (TRU has a positive effect on PU), and none of the standard errors (S.E.) of estimated parameters were negative, which indicates that the results of the path analysis are valid [97]. In general, six out of eight hypotheses were supported by the data. The detailed findings are discussed in Section 6.

### 5.3. Moderation Effect

We conducted a multiple regression analysis using IBM SPSS Statistics 25 (IBM, Armonk, NY, USA) to examine whether the construct DPR plays the role of moderator in the effect of PR on TRU, and this hypothesis was described as hypothesis H6.

In order to exclude some other factors that may affect the regression results, we firstly set several control variables, including sex (1 = “male”, 2 = “female”), age (1 = “≤20 years old”, 2 = “21–40 years old”, and 3 = “>40 years old”) and education background (1 = “under college”, 2 = “college or university”, and 3 = “graduate school”). Sometimes, sex can be an unstable factor that can affect consumers’ trust, as females may show more trust in a peer-recommended product than males [100]. Additionally, sex differences should be treated seriously in the field of perceived risk [101]. Therefore, the variable “sex” was controlled in this research. Similarly, in the framework of TAM, age and education background may influence consumers’ perceived risk and behavioral intention [102]. Specifically, younger research participants or research participants who are highly educated may be easier to accept a fresh method of treatment. Therefore, these two factors were controlled for too.

The results of the regression analysis are shown in Table 6. We set four regression models to observe the moderation effect of DPR (**H6:** DPR significantly moderates the relationship between PR and TRU). After setting TRU as the only dependent variable, the control variables (sex, age, and education background) were entered firstly (Model 1). Then, the independent variable PR was entered at step 2 (Model 2); the next step was to add the moderator DPR into the third regression model as a reference (Model 3). Last, an interaction term (PR × DPR) was entered to examine **H6** (Model 4). Additionally, the PR and DPR data were mean-centered to exclude possible multicollinearity before producing this interaction term. The results showed a significant interaction effect of PR and DPR on TRU (β = 0.097, *p* < 0.1), and the F change for △R^2^ was significant as well (F = 2.826, *p* < 0.01). Additionally, the independent (PR) and moderated (DPR) variables were both significant (β = −0.324, *p* < 0.01; β = 0.474, *p* < 0.01). To sum up, DPR showed a moderating effect on the relationship between PR and DPR. In other word, **H6** was supported as well as **H5**.

**Figure 3:**
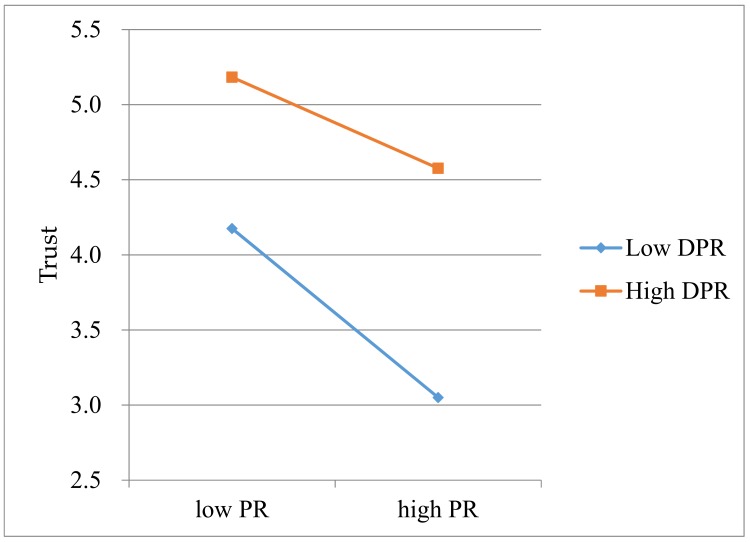
Interactive effects of PR and DPR on TRU.

To figure out how DPR moderates the influence path “PR → DPR”, an interaction plot, in which the interaction was plotted with a standard deviation above (+1 SD DPR) and below (–1 SD DPR) the mean as a high level and a low level [103], was drawn to reveal the interactive effect of PR and DPR on TRU [104]. As shown in Figure 3, PR negatively affects TRU whether PR is at a low or high level, but the influence degree is much weaker at a high level than that at a low level. Specifically, when DPR is at a high level, TRU is less negatively affected by PR, and TRU does not decrease rapidly as PR increases. In contrast, when DPR is at a low level, TRU is more negatively affected by PR, and TRU will decrease rapidly as PR increases. Therefore, the moderating effect of DPR has been certified.

## 6. Discussion

The aim of this research was to investigate how perceived risk affects patient’s behavioral intention to use online medical websites using trust and the moderating effect of the doctor–patient relationship as variables. We conducted a structural equation analysis to solve the first question and a regression analysis for the second one. We presented eight hypotheses, and six of them were supported by the analysis.

As an authoritative research framework in the field of individual choice, the technology acceptance model was adopted as a basic framework and was an important part of our research model. The structural equation analysis results revealed that apart from **H2a** (PU is positively related to patients’ ATT), all hypotheses in this framework were supported, which indicates that although almost 30 years have passed since its development, Davis’s (1989) TAM framework is still suitable for assessing patients’ choice of medical services [33]. Therefore, we suggest that researchers should consider TAM as an important reference if they want to explore the key factors affecting patients’ intention to choose a specific medical product or service. Furthermore, since PU did not show a significant effect on ATT but affected BI directly, we consider that consumers’ decision-making processes have been simplified as consumers have become used to online purchasing. The usefulness of a product may motivate consumers to form a behavioral intention directly. Therefore, ATT can be omitted when researchers use the framework of TAM in the future, as in the work of Chow et al. (2012) [68,69,70].

In particular, ATT (β = 0.391, *p* < 0.01) and PU (β = 0.491, *p* < 0.01) are two important factors that can positively influence patients’ BI directly. Additionally, PEOU has a strong positive effect on PU (β = 0.940, *p* < 0.01) and ATT (β = 0.847, *p* < 0.1). Although these paths have been certified several times [65,68,69], some researchers have reported opposite results [61,70]. Additionally, PU did not show a significant influence on ATT, which is different from the results of Davis and Venkatesh (1996) [34]. We believe that the research models of different products are customized for specific products when researchers study individual choice. Therefore, although TAM provides a general explanation for individual choice in the field of consumer adoption, the results generated by the different models still show differences. This is a reason why this paper designed a modified TAM based on the community characteristics of OMW users in China to explore the key factors influencing patient use of OMWs. According to the discussion, our suggestion is that it is very important for medical service providers to ensure patients find OMWs useful and easy to use. To do that, they need to transform the marketing strategy from “educated by consumers” to “educating consumers”. Under the current situation, it is difficult for new consumers to use OMWs with no problems, especially those who cannot use the Internet skillfully. Therefore, the most visible place on the OMWs should be the operations guide video, and many success cases should be displayed in order to prompt consumers to believe that OMWs are useful and convenient for them.

In contrast to our expectations, TRU was not shown to have a significant effect on PU, but TRU is still an important factor in the whole influence process as it can affect PU, ATT, and BI indirectly through PEOU (β = 0.798, *p* < 0.01). Therefore, it is crucial for medical service providers to retain the trust of consumers in their products. Although many mainstream providers have increased their quality of services, they are not simply trusted enough by many consumers due to worries about security of money and personal information [105]. In other words, patients’ trust in OMWs will decrease if they perceive that financial risk, physical risk, or privacy risk is present. Not surprisingly, our data certified that patients’ PR is negatively related to their trust in OMWs (β = −0.552, *p* < 0.01). Therefore, patients’ PR should be inhibited during the process of using OMWs. We have several suggestions for medical service providers. Firstly, they should allow patients to use modes of payment that are widely trusted by consumers, like Zhifubao and Wechat (The most widely used online wallet in China), to reduce their perceived financial risk. Secondly, doctors’ licenses should be shown to patients before they decide to accept a diagnosis online, and they should be informed that the health insurance will pay if they are misdiagnosed. In this way, patients’ perceived physical risk will be decreased, indirectly increasing their intention to use OMWs. Thirdly, in order to reduce patients’ perceived privacy risk, patients should be allowed to use pseudonyms unless they need to buy prescription drugs online. Finally, our data certified that the doctor–patient relationship is a significant moderator that influences the effect of PR on TRU. Additionally, PR’s negative effect will be strengthened if the DPR is at a low level, while this kind of negative influence can be weakened if there is a good relationship between patients and doctors. Therefore, we suggest that the OMWs should provide a thorough responsive feedback mechanism. Doctors can be sued if they do not show a good attitude when communicating with patients, and complaints should be published by the platform if they are found to be valid. Anyway, medical service providers should do their best to make patients feel that they can build a good (or at least equal) relationship with doctors.

This study also has several limitations. First, the independent variable perceived risk had only three aspects (perceived financial risk, perceived physical risk, and perceived privacy risk) in this research; however, research participants may perceive another type of risk which has not been identified during the use of OWMs. However, it is difficult to conduct longitudinal research to trace the subsequent feelings of our respondents, although we may explore more detailed findings if we can collect more samples. Lastly, researchers should pay attention to the fact that it is conceivable that responses from online participants could differ from offline participants due to effects of the mode of administration. In other words, there may be bias between the data collected online and the data collected offline [106]. A future field experiment may to help eliminate this kind of bias and make the results of the empirical analysis more rigorous. Future research should involve (1) subsequent analysis to determine the key factors affecting patients’ continued behavioral intention towards OMWs, (2) a meta-analysis to prove that the moderating effect of DPR is widespread, and (3) an exploration of the reasons why DPR plays a moderating role (the culture, for example) in the influence process.

## 7. Conclusions

In this research, we constructed a modified TAM based on the characteristics of OMWs and the TAM framework to examine how PR affects BI through TRU, PEOU, PU, and ATT. We also examined the moderating effect of DPR. The results of the data analysis reveal that PR is negatively related to TRU. TRU has a positive effect on PEOU but has no significant effect on PU. PEOU has positive effects on both PU and ATT. PU is positively related to BI but has no significant effect on ATT. ATT is also positively related to BI. Finally, DPR can significantly moderate PR’s effect on TRU. In general, patients’ PR can negatively affect their BI through their TRU, PEOU, PU, and ATT, while DPR can moderate the negative influence of PR on TRU.

Based on the results of the data analysis, we suggest that it is important for OMW providers to improve patients’ trust and perceived risk in order to increase their behavioral intention, and we give some detailed methods for further innovation in the discussion section. Additionally, the vitality of OMW providers will be promoted as the consumers’ behavioral intentions improve. Finally, we acknowledge that there is still room for improvement in this study. We may modify the survey or develop a new one to fit the habits of Chinese consumers. Future research will include another empirical study including more samples and more key influence factors to explain users’ adoption of OMWs more effectively.

From a theoretical perspective, the current literature cannot reveal the moderation mechanism of the doctor–patient relationship that has been shown by this research. Therefore, we provide a valuable reference for future research in the field of individual choice. From a practical perspective, our research indicates that five key factors affect patients’ adoption of OMWs. Additionally, we have provided several meaningful suggestions for medical service providers to attract more users and maintain their existing users. We believe that OMWs could provide better services for patients with poor medical conditions, especially in rural areas, and promote the rational allocation of medical resources and medical equity.

## Figures and Tables

**Table 1:** Demographic characteristics.

Item	Category	Number	Percent (%)
Gender	Male	99	40.41%
	Female	146	59.59%
Age	≤20 years old	42	17.14%
	21–40 years old	172	70.20%
	>40 years old	31	12.65%
Education background	Less than Bachelor’s degree	52	21.22%
	Bachelor’s degree	168	68.57%
	Master’s or Doctoral degree	25	10.21%

**Table 2:** Factor Loading, C.A., AVE, and CR.

Construct	Item Code	Factor Loading	C.A.	AVE	CR
Doctor–Patient Relationship (DPR)	DPR1	0.734	0.85	0.61	0.89
	DPR2	0.812			
	DPR3	0.779			
	DPR4	0.782			
	DPR5	0.792			
Trust (TRU)	TRU1	0.874	0.87	0.73	0.89
	TRU2	0.824			
	TRU3	0.859			
Perceived Risk (PR)	PPriR	0.758	0.89	0.68	0.87
	PFR	0.863			
	PPhyR	0.851			
Perceived Usefulness (PU)	PU1	0.722	0.85	0.56	0.84
	PU2	0.702			
	PU3	0.773			
	PU4	0.803			
Perceived Ease of Use (PEOU)	PEOU1	0.647	0.80	0.58	0.85
	PEOU2	0.852			
	PEOU3	0.857			
	PEOU4	0.671			
Attitude (ATT)	ATT1	0.781	0.85	0.53	0.77
	ATT2	0.661			
	ATT3	0.735			
Behavioral Intention (BI)	BI1	0.712	0.78	0.53	0.77
	BI2	0.595			
	BI3	0.851			
The Whole Questionnaire			0.86		

Note. C.A. = Cronbach’s alpha; AVE = average variance extracted; CR = composite reliability.

**Table 3:** Means, standard deviations, and correlations of the constructs.

Construct	Mean	SD	DPR	TRU	PR	PU	PEOU	ATT	BI
DPR	4.935	1.162	**0.781**						
TRU	4.246	1.336	0.548 ***	**0.854**					
PR	4.793	1.141	−0.241 ***	−0.404 **	**0.825**				
PU	4.746	1.228	0.395 ***	0.611 **	−0.199 **	**0.748**			
PEOU	4.664	1.184	0.412 ***	0.589 **	−0.165 **	0.670 **	**0.762**		
ATT	4.592	1.325	0.352 ***	0.601 **	−0.277 **	0.668 **	0.648 **	**0.728**	
BI	4.510	1.192	0.060	0.293 ***	−0.121 *	0.450 ***	0.316 ***	0.507 ***	**0.728**

Note. DPR = doctor–patient relationship; TRU = trust; PR = perceived risk; PU = perceived usefulness; PEOU = perceived ease of use; ATT = attitude; BI = behavioral intention; AVE = average variance extracted; SD = standard deviation; ** *p* < 0.01.

**Table 4:** Recommended and actual values of fit indices.

Fit Indices	CMIN/DF	GFI	AGFI	PGFI	CFI	NFI	PNFI	IFI	TLI (NNFI)	RMSEA
Recommended Value	<3	>0.90	>0.80	>0.50	>0.90	>0.90	>0.50	>0.90	>0.90	<0.08
Actual Value	1.742	0.904	0.869	0.663	0.958	0.908	0.736	0.959	0.948	0.055

Note. CMIN/DF = ratio between chi-squared and degrees of freedom; GFI = goodness of fit index; AGFI = adjusted goodness of fit index; PGFI = parsimony goodness of fit index; CFI = comparative fit index; NFI = normed fit index; PNFI = parsimony normed fit index; IFI = incremental fit index; TLI (NNFI) = Tucker–Lewis index (non-normed fit index); RMSEA = root mean square error of approximation.

**Table 5:** Model path analysis.

The Hypothesis	Path Coefficient	S.E.	Support
H1: Attitude → Behavioral intention	0.391 ***	0.093	Yes
H2a: Perceived usefulness → Attitude	n.s.	0.163	No
H2b: Perceived usefulness → Behavioral intention	0.491 ***	0.082	Yes
H3a: Perceived ease of use → Perceived usefulness	0.940 ***	0.172	Yes
H3b: Perceived ease of use → Attitude	0.847 ***	0.227	Yes
H4a: Trust → Perceived usefulness	n.s.	0.100	No
H4b: Trust → Perceived ease of use	0.798 ***	0.060	Yes
H5: Perceived risk → Trust	−0.552 ***	0.114	Yes

Note: S.E. = standard error; n.s. = not significant; * *p* < 0.1; ** *p* < 0.05; *** *p* < 0.001.

**Table 6:** Results of the regression analysis.

Variables	TRU			
	Model 1	Model 2	Model 3	Model 4
Sex	−0.021	−0.004	0.039	0.038
Age	0.116 *	0.082	0.025	0.032
Education Background	−0.169 ***	−0.104 *	−0.055	−0.056
PR		−0.378 ***	−0.280 ***	−0.324 ***
DPR			0.473 ***	0.474 ***
PR × DPR				0.097 *
R square	0.043	0.181	0.385	0.392
△R^2^	0.031	0.167	0.372	0.377
F change for △R^2^		40.264 ***	79.302 ***	2.826 ***

Note. TRU = trust; PR = perceived risk; DPR = doctor–patient relationship; △R^2^ = Adjusted R square; *** *p* < 0.01; ** *p* < 0.05; * *p* < 0.1.

## Appendix A. Questionnaire

This questionnaire is being used to identify the key factors affecting patients’ behavioral intention to use online medical websites like Chunyuyisheng and Haodaifu. Please confirm that you are an adult (≥18 years old) who has experience with using online medical websites before filling out this questionnaire. Thank you.

We inform you that there are no right/wrong or good/bad answers in this questionnaire; all the data we collect are confidential and for academic use only.

**Part 1. Measurement Scales and Items**

Note. This is a 7-point Likert scale. There are 7 numbers (1, 2, 3, 4, 5, 6, 7) below each item. Among them, “1” means “strongly disagree” while “7” means “strongly agree”. Please circle the number that best fits your real feelings on the item to represent the degree to which you agree or disagree with the statement of that item.

**Doctor–Patient Relationship** (DPR; adapted from Chung-Feng Liu et al. (2013))

**DPR1**: The doctors give you (the patient) enough time.						
1(strongly disagree)	2	3	4	5	6	7(strongly agree)
**DPR2**: The doctors listen to you.						
1(strongly disagree)	2	3	4	5	6	7(strongly agree)
**DPR3**: The doctors explain tests and treatments.						
1(strongly disagree)	2	3	4	5	6	7(strongly agree)
**DPR4**: The doctors involve you in decisions about your care.						
1(strongly disagree)	2	3	4	5	6	7(strongly agree)
**DPR5**: The doctors treat you with care and concern.						
1(strongly disagree)	2	3	4	5	6	7(strongly agree)

**Perceived Risk (PR) 1:** Perceived Privacy Risk (PPriR; adapted from Hajli and Lin (2016))

**PPriR1**: I am concerned that OMWs are collecting too much personal information about me.						
1(strongly disagree)	2	3	4	5	6	7(strongly agree)
**PPriR2:** I am concerned about the privacy of the personal information that OMWs captures about me.						
1(strongly disagree)	2	3	4	5	6	7(strongly agree)
**PPriR3:** I suspect that my privacy is not well protected by the online medical websites.						
1(strongly disagree)	2	3	4	5	6	7(strongly agree)
**PPriR4:** I’m worried that unknown third parties will access my personal information on the online medical websites.						
1(strongly disagree)	2	3	4	5	6	7(strongly agree)

**Perceived Risk (PR) 2**: Perceived Financial Risk (PFR; adapted from Featherman and Pavlou (2003))

**PFR1:** When transferring money on the Internet, I am afraid that I will lose money due to careless mistakes such as wrong input of account number and wrong input of the amount of money.						
1(strongly disagree)	2	3	4	5	6	7(strongly agree)
**PFR2:** When transaction errors occur, I worry that I cannot get compensation from banks.						
1(strongly disagree)	2	3	4	5	6	7(strongly agree)

**Perceived Risk (PR) 3:** Perceived Physical Risk (PFR; recomposed from Hajli and Lin (2016))

**PFR1**: I am concerned that the treatments from doctors of online medical websites will negatively affect my health.						
1(strongly disagree)	2	3	4	5	6	7(strongly agree)
**PFR2**: I am concerned that the treatments from doctors of online medical websites are not safe.						
1(strongly disagree)	2	3	4	5	6	7(strongly agree)
**PFR3**: I am concerned that the drugs bought from online medical websites are not safe.						
1(strongly disagree)	2	3	4	5	6	7(strongly agree)

**Trust** (TRU; adapted from Lee (2004))

**TRU1**: Online medical websites are trustworthy.						
1(strongly disagree)	2	3	4	5	6	7(strongly agree)
**TRU2**: Online medical websites keep their promises and commitments.						
1(strongly disagree)	2	3	4	5	6	7(strongly agree)
**TRU3**: Online medical websites have the patient’s best interest in mind.						
1(strongly disagree)	2	3	4	5	6	7(strongly agree)

**Perceived Usefulness** (PU; adapted from Venkatesh and Davis (2000))

**PU1**: Using online medical websites improves my performance in my work and life.						
1(strongly disagree)	2	3	4	5	6	7(strongly agree)
**PU2**: Using online medical websites can increase my productivity.						
1(strongly disagree)	2	3	4	5	6	7(strongly agree)
**PU3**: Using online medical websites can solve my health problems.						
1(strongly disagree)	2	3	4	5	6	7(strongly agree)
**PU4**: I find online medical websites to be useful for my life.						
1(strongly disagree)	2	3	4	5	6	7(strongly agree)

**Perceived Ease of Use** (PEOU; adapted from Venkatesh and Davis (2000))

**PEOU1**: My interactions with online medical websites are clear and understandable.						
1(strongly disagree)	2	3	4	5	6	7(strongly agree)
**PEOU2**: Interacting with online medical websites does not require substantial mental effort.						
1(strongly disagree)	2	3	4	5	6	7(strongly agree)
**PEOU3**: I find online medical websites easy to use.						
1(strongly disagree)	2	3	4	5	6	7(strongly agree)
**PEOU4**: I can easily solve my problems through online medical websites.						
1(strongly disagree)	2	3	4	5	6	7(strongly agree)

**Attitude** (ATT; adapted from Venkatesh et al. (2003))

**ATT1**: Using online medical websites is a good idea.						
1(strongly disagree)	2	3	4	5	6	7(strongly agree)
**ATT2**: I like using online medical websites.						
1(strongly disagree)	2	3	4	5	6	7(strongly agree)
**ATT3**: I am willing to solve my health problems with online medical websites.						
1(strongly disagree)	2	3	4	5	6	7(strongly agree)

**Behavioral Intention** (BI; adapted from Venkatesh et al. (2003))

**BI1**: I intend to use online medical websites in the future.						
1(strongly disagree)	2	3	4	5	6	7(strongly agree)
**BI2**: I predict that I will use online medical websites in the future.						
1(strongly disagree)	2	3	4	5	6	7(strongly agree)
**BI3**: I plan to use online medical websites in the future.						
1(strongly disagree)	2	3	4	5	6	7(strongly agree)

**Part 2. Personal Information Card**

1. Choose your gender from the two options below, please.		
A. Male		B. Female
2. Choose your age from the three options below, please.		
A. ≤20 years old	B. 21–40 years old	C. >40 years old
3. Choose your education background from the three options below, please.		
A. less than Bachelor’s degree	B. Bachelor’s degree	C. Master’s or Doctoral degree
